# Permissibility of Multifetal Pregnancy Reduction from The Shiite Point of View

**DOI:** 10.22074/ijfs.2016.5088

**Published:** 2016-11-01

**Authors:** Atefeh Zabihi Bidgoli, Faezeh Azimzadeh Ardbili

**Affiliations:** 1 Department of Family Law, Faculty of Law, Imam Sadiq University, Tehran, Iran; 2 Department of Theology and Law, Imam Sadiq University, Tehran, Iran

**Keywords:** Fetal Reduction, Multifetal Pregnancy, Embryo

## Abstract

**Background:**

Advancements in medical technology have significantly increased the possibility of successful infertility treatment. Medical interventions in the initial process of pregnancy that intend to increase the chances of pregnancy create the risk of multifetal pregnancies for both mothers and fetuses. Physicians attempt to reduce the numbers of fetuses in order to decrease this risk and guarantee the continuation of pregnancy. The aim of this paper is to understand the Shiite instruction in terms of the risks multifetal pregnancies have for fetuses and if it is permissible to reduce the numbers of fetuses. An affirmative answer will lead to the development of Islamic criteria for reduction of the number of embryos.

**Materials and Methods:**

This analytical-descriptive research gathered relevant data as a literature search. We reviewed a number of Islamic resources that pertained to the fetus; after a description of the fundamentals and definitions, we subsequently analyzed juridical texts. The order of reduction was inevitably determined by taking into consideration the rules that governed the abortion provisions or general juridical rules. We also investigated the UK law as a comparison to the Shiite perspective.

**Results:**

The primary ordinance states that termination of an embryo is not permitted and is considered taboo. However, fetal reductions that occur in emergency situations where there is no option or ordinary indication are permitted before the time of ensoulment. The goal of reduction can be chosen from different ways.

**Conclusion:**

According to Shiite sources, fetal reduction is permitted. Defective fetuses are the criteria for selective reduction. If none are defective, the criteria are possibility and facility. But if the possibility of selection is equally for more than one fetus, the criterion is importance (for example one fetus is healthier).

## Introduction

Due to advancements in technology and the emergence of innovative technology in the field of infertility, currently few spouses are unable to fulfill their aspirations to produce children. Although these methods are efficient, they raise many juridical questions. Answering these questions impacts the quality and methods of treatment. 

One of these problems is that physicians encounter multiple pregnancies. This possibility increases as a result of ovarian stimulation and infertility treatments. Recent decades show these statistical trends where, from 1980 to 2009, only in America have the numbers of twins or triplets increased to 76%, an increase from 18.9 to 33.3 for each 1000 births (1). 

A risk of multiple pregnancies exists both for the mother and the fetuses. Hence, selective reduction of the fetuses is a strategy suggested by physicians. The aim of this paper is to investigate the permissibility or impermissibility of fetal reduction from the Shiite view. However, it is necessary to define selective reduction or multifetal pregnancy reduction (2). The meaning of selective reduction is the removal of one or several fetuses in a pregnancy that has greater than one fetus with the intent to achieve a pregnancy with twin fetuses or one fetus. In this step, the physician aborts the surplus fetuses that have been implanted to the patient’s womb to increase her chances of fertility or these fetuses formed because of stimulating drugs that caused the release of several ovules. 

One method has been used for more than two decades to prevent morbidity (3). Usually, this method requires injection of potassium chloride (KCl) into the heart of one or more fetuses (4), among other methods. Before investigating the ordinance of different features of reduction, it is necessary to clarify the general ordinance that pertains to the Shia regarding reduction of fetuses. 

## Materials and Methods

This analytical-descriptive research was undertaken at Imam Sadiq University in October, 2015. We reviewed a number of Islamic resources about the fetus, its life, and conducted a bibliography study on the basis of medical and legal resources. The resources included books, articles, and internet sources. The study was based on comparative and annalistic studies. 

This analytical-descriptive research gathered relevant data in the form of a literature search. After a description of the fundamentals and definitions, juridical texts were subsequently analyzed and the order of reduction inevitably determined by taking into consideration the rules that governed the abortion provisions or general juridical rules and principles which exist in Islamic sources. Finally, we compared the Shiite perspective with the UK law. 

In some cases reduction is permitted according to Islamic instructions. In the current research we sought to enumerate the cases permitted by Islam. We have determined the criteria for selection of the fetuses for the reduction process by investigating the primary ordinance of Islamic jurisdiction. The primary ordinance is introduced beside the secondary ordinance. It is an ordinance that law makers will generate according to the discretion and the loss that has originated in the issue, such as prohibition of wine or murdering. The secondary ordinance is an ordinance issued when there is an emergency, loss, or necessity such as removal of the ordinance of prohibition of wine at the time of an emergency (5). The second ordinance is studied according to the relationship of the conditions that the reduction has suggested. 

## Results

A study of the permissibility of multifetal pregnancy reduction is dependent upon investigation into the primary and secondary ordinances of fetal reduction followed by the jurisprudence position. In each case of embryo reduction the conditions should be defined. Criteria for selection in fetal reduction and conducting a comparative study of the Shiite perspective versus UK law were the final steps to remove possible legal gaps in this area. 

### Investigation about the primary and secondary ordinances of fetal reduction

Fetal reduction and abortion have one consequence-cessation of a life. However in fetal reduction, unlike abortion, the pregnancy is not terminated. Hence it seems that specifying the first and second ordinances of abortion would be sufficient. Hence, we can transmit this ordinance to fetal reduction which is a new phenomenon. 

### Primary ordinance

In view of Imamiyah jurisdiction, the fetus is a respectful existence. The Quran as well as the Prophet and Imams (pbut) behavior that are defined as "tradition, logical reason, and consensus" state that abortion is illegal. 

According to Imamiyah jurisdiction, even "blood money" is defined for the seed of a human being (sperm) prior to entry into a woman’s womb (6,7). Then, this sum of money differs when the fetus grows in the mother’s womb where five stages (seed, clot, lump, bones, and bone clothed by flesh) are defined (7). Therefore, abortion is not permitted in Islam. 

According to verses 12 to 14 of Sura Al-Mumenoon in the Quran, fetal life includes two basic stages: i. From the beginning of combining the ovule with the sperm until ensoulment (the time when the fetus possesses a sprit) and ii. From ensoulment until birth. There is no unique belief about the time when a fetus possesses a spirit. In the view of most jurisdictions, when the embryo is in the fourth month, he or she possesses a sprit (8). 

In the first stage before ensoulment the fetus is similar to a plant rather than a human being. However, its killing is taboo according to narratives. In the second stage, killing of the fetus is also taboo. 

Although there is no difference among the Shia about the illegality of an abortion after ensoulment (9), its reasons are various. Some jurists, according to Surah Al-Anaam verse 151 of the Quran (8,10), consider the fetus to be human. In this case, an abortion is regarded as a murder and meets the criteria for retaliation (11,13). In this verse Allah says: "You do not kill the soul which God has sanctifiedexcept in the course of justice". 

Others deny this justification and retaliation against abortion. They believe that abortion is illegal because according to certain narratives (14), the blood money of a fetus after ensoulment is equal to a human. This group claims that the mother’s and fetus’s lives are the same and there is no preference between them. 

Other justifications have been mentioned about the illegality of abortion. Each contains impediments. The authors (as some jurists have said) believe that the mentioned reasons are not acceptable for proving equality between the mother’s and fetus’s lives. This statement is also confirmed by the fact that jurists prefer the mother's life when the her life conflicts with an abortion. In this case the abortion is permitted. If the value of the fetus’s and the mother’s lives are equal, there is no rationale for this judgment. On the one hand, what is said in the Quran cannot be true about a fetus since it is talking about murdering a human. On the other hand, the equality of blood money for the mother and fetus cannot prove the similarity between a mother and a fetus. Therefore the only justifications about the illegality of abortion are what our Imams have said in the narratives. After all, abortion is illegal and taboo in Shia where there is a consensus. Abortion is taboo is enough to prove that despite specified cases, abortion is not permissible in other cases. 

Logical thinking also prohibits oppression and it is likely to say that abortion is a type of oppression. Abortion is a form of aggression toward a person who cannot defend himor herself (15). According to Islam, the fetus is respected from the beginning because of its genesis and it enjoys the right to life. Agreement of the spouse is not enough for fetus reduction. Creation of the fetus is the result of parental sexual flow and not enough to allow them to destroy the fetus after reproduction. 

Although fetus reduction in Islam is prohibited, some cases known as secondary ordinances are permitted as discussed below. 

### Secondary ordinance

Although abortion is taboo in Islam, it is permitted in cases of emergency, hardship, and loss. Islamic jurisdiction has defined two specific cases in which abortion is permitted. First, when maintaining the fetus endangers the mother’s life. This case is known as "tazaahom" means the possibility of gathering two ordinances simultaneously while they are in existence but there is an inability to obey both ordinances (16). The ordinance "necessity to protect the mother’s life" contrasts the ordinance of "necessity to protect the fetus’s life", as the mother’s life is more important than the fetus’s life. Therefore the mother’s life is preferred (17) because the mother is a real human being whereas the fetus is potentially a human. This preference is enough to save the mother’s life. Likewise, crimes against the mother deserve "retaliation" whereas those against the fetus deserve "blood money". This is the reason that the mother's life is preferred (15). Other reasons, for example, consider abortion to be a means of self-defense for the mother. According to the defense theory, saving the mother’s life is preferred, because if the mother dies, the fetus will also die (18,19). 

Of note, in cases where the survival of the fetus results in birth defects or intolerable pain for the mother and protecting the fetus outside of the womb is not possible, the following rules (a and b) permit abortions as tolerating this loss is difficult for the mother (20). Rule a states: "juridical regulation of negating difficulties and troubles". This rule says that whatever causes a problem or risk is negated by Islamic law makers. The best evidence comes from verse 87 of Surah AlMumanoon in the Quran: "There is no difficulty in your religion" (21). 

Rule b is the "rule of negation of loss". The contingency of this act is that no man is permitted to cause loss to others in order to prevent his own loss. It is not permissible to cause loss to him to prevent loss to others (22). 

The second case occurs when the fetus is deformed and defective. In such cases, since the birth of a deformed baby will create a severe hardship for the parent, abortion is permitted before ensoulment. However another perspective exists. According to this view, although tolerating an illness is difficult, it is a "test" for the parents and the fetus. According to Islamic view, Allah tests his slaves (verse 145 of Surah Al-Baqara) (23). Then, as killing patients and defective people is not permitted, killing a defective fetus is also not permissible. 

Permission to kill the fetus in such cases is limited to the stage where the fetus does not have a spirit. After ensoulment, as this act considers the rights of all assignees, one cannot derive a benefit for someone (mother) and cause loss for others (fetus) (15,20,24). Therefore, it should be considered that in all cases where selective reduction is permitted, this permission is limited to the stage where the fetus does not possess a spirit. After this stage, fetus reduction is illegal with the exception of tazaahom. 

By taking into consideration the abovementioned, if cases of multiple fetuses where continuation of pregnancy causes any hazard for the mother, the fetus reduction is considered secondary and it is not necessary to obtain the husband’s permission. 

To the extent possible, the number of transferred fetuses should be minimal so that selective decrease of fetuses will be unnecessary. In order to prevent multifetal pregnancy, the number of transferred fetuses should be minimal or small doses of medication (stimulating drugs that caused the release of several ovules) should be prescribed. However this will decrease the chances of fertilization. For example, if after ovary stimulation, more than three follicles have matured, the cycle should be cancelled at both ovaries to prevent multiple pregnancies. In assisted reproductive technology (ART) implantation, fewer fetuses are recommended. In elective single embryo transfer (eSET) it is recommended to choose only one high quality fetus. Unfortunately, the high cost of infertility treatment and small success rate result in the implantation of multiple fetuses in the mothers’ womb to increase the chances of childbirth (25). In this regard, to counteract, some countries have approved acts in which under any circumstances, it is not permissible to transfer more than one fetus by the physicians (26). However, the duration of treatment following the implantation of one fetus and high treatment cost encourage the implantation of more fetuses. 

Even in cases where the transfer of more than one embryo to the mother’s womb is justifiable, it is clear that most mothers prefer not to be faced with reduction of the fetuses. In such cases the mothers may encounter extreme psychological problems because of the length of time spent waiting to have children (27). In such cases, embryo implantation should be completely controlled in a way that the possibility of multiple pregnancies decreases. When fertility centers compete to attract more patients and increase the rate of fertility and their own success, it is necessary to prevent them from misusing patients by persuading them to implant more embryos, after which reduction of the fetuses becomes necessary. 

### Defining the position of jurisprudence for each case of embryo reduction by taking into consideration the conditions 

Considering the risk of continuation of multiple pregnancies for fetuses, jurisprudence have posed the following assumptions (28).

1 The conditions that reduction of embryos will not endanger the mother’s life for continuation of pregnancy and the fetuses do not have any defect and no damage entreats them.
  In these cases, fetal reduction is illegal. Physicians and well-informed parents are persecuted for their civil and penal responsibilities. Hence, reduction of fetuses in cases where there is no risk for the mother or the fetus is not permitted. The general ordinance of fetus reduction is considered. As previously stated in the primary ordinance, according to Islam, the fetus is respected from the beginning of its genesis and it has the right to live. Therefore abortion and reduction are illegal.
2 During the pregnancy, the medical test shows the existence of abnormalities. In this case, reduction is approved in some fetuses. In such cases, the fetus deficiency is the reason for reduction. After approval of damage and hardship for the parents, an abortion is permitted according to the criterion of the secondary ordinance because the juridical evidences of prohibition of abortion do not include these cases according to some jurisprudence (15).
3 Continuation of multiple pregnancies is dangerous for the fetuses. According to resources, cases where a deficiency in the fetus is the reason for reduction are defined as "selective termination of pregnancy" whereas cases that the reason of reduction is the risk for mothers and fetuses are called "fetal reduction of multiple pregnancies" (29).

Cases where reduction of the fetus is chosen as a guarantee for healthiness of the pregnancy have different ordinances according to whether multiple pregnancies occurred naturally or by implantation and the physician. Natural multiple pregnancies occur when the number of fertilized ova are more than one or when there is a mutation in embryo’s cells during division. Artificial multiple pregnancy occurs when the physician decides to implant several fetuses as a cure for infertility. The physicians, after receiving permission from the patient, stimulate the ovary with medications or implant several fetuses into the womb. Then, permission of reduction of the fetuses under two conditions is assumed. 

For example, multiple pregnancies are natural and preservation of surplus embryos endangers the lives of all embryos. Here, keeping each embryo conflicts (in the exact meaning of tazaahom) with the lives of the others. Because the situation is equal for all, the priority of one embryo over the others is considered “preference without any logical reason”. Preference without any logical reason means that one thing is preferred over the other in instances where both have equal features and without any special goal. This is an impossible issue. Most philosophers assume this is a reasonable rule and obvious, in a way that obviousness is evident such as the "premise of unity is half of two" (30). In such cases no embryo is preferred over another. In such cases one cannot delay and endanger the life of all fetuses. So, referring to the jurisprudence rule of "Al-maysoure la yasqoto bel-masoure" we cannot eliminate all embryos. Inevitably we should act according to an acceptable criterion. According to this rule, when someone is obliged to do something, he or she should do it according to his or her capabilities. It is mentioned as "what one cannot do completely, he should not abandon it completely" (31). In another interpretation, the lawmaker orders to something that is composed of several parts, and some parts have obstruction and some parts do not. In cases where the parts do not have any obstruction, they should not avoided (32). 

In the second situation where multiple gestation is artificial and maintaining all the embryos would endanger the lives of all embryos. In this case, continuing with the pregnancy is a conflict between the mother’s life and multiple synthetic pregnancies, which has emerged as a result of the decision of the parents. This can induce doubt that this agreement is subject to the legal rule of "emergency optionally does not negate arbitration". The purpose of this rule is that of someone who is obliged engages in a taboo activity, in a way that he or she cannot act according to the rule. Regarding that his inability to perform the task that has been caused by incorrect disposal (conscious possession), his or her responsibility will not decrease (21). In this way, by an incorrect decision, the mother’s situation is so that she cannot preserve her fetus. Thus, fetal reduction is not possible for her. If she does so, she will be prosecuted. We can say that conscious multiple pregnancies will put her in a situation that cannot defend herself against the emergency of abortion. 

In response to this question it is accepted that "regarding legal reasons and principles, if the situation of a person is a result of an adverse selection, this situation puts him out of the emergency, whereas in such circumstances it is impossible to have a bad choice" (28). It should be added that, according to the aforementioned rule, the distinction between "those emergency situations that cause irresponsibility" and "avoidance willfully" is that the person with the bad choice cannot fulfill her duties, whereas in multiple pregnancies the physician and parents try to achieve the best results for the treatment of infertility. 

In this case, as with the first one, reduction of the fetuses is permitted unless the mother is in a position in which she is forced to reduce the fetus because of her previous adverse possession. 

Even when the parents demand implantation of more embryos to enhance the chances for fertility, it is the responsibility of the physician to implant fewer embryos to avoid fetal reduction. Implantation of surplus embryos by the physician is the exact case for adverse selection that results in prohibition of the fetus reduction. 

Of note, according to the fact that the result of both fetal reduction and abortion is the same (fetal deaths), fetal reduction (which is fully justified medically) is legally permitted in countries where abortion is not illegal, even without any medical reason. 

### Criterion for selection in fetal reduction

The reasons for permission of reduction of the fetuses include: deficiency, importance, ease, random choice, parents’ right, and governmental ordinance. 

According to the deficiency criterion in cases where reduction of the fetus is necessary, if a fetus is defective, this fetus would be the goal for reduction. This case has been discussed previously. 

Importance. In terms of the ordinance that each fetus is preserved by reduction of another fetus, these ordinances conflict (in the exact meaning of tazaahom) with each other because a pregnant women should choose one of two ways: i. Keep all fetuses which ultimately results in the deaths of all the fetuses or ii. Reduction of some fetuses that ends in the deaths of those fetuses and continuation of pregnancy for the other fetuses. 

When there is a conflict (in the exact meaning of tazaahom) between two or more discretions, it is important to consider the most important discretion (33). In order to enforce the rule of the most important discretion it is supposed that if it is possible to predict the importance of survival of one fetus over the others, the protection of that fetus is preferred (28). In cases that there is a considerable defect or deformity in the fetus compared to the others, the criterion is to reduce this fetus and keep the others. However the meaning of defection is not those defections that lawmakers have issued regarding the rule of reduction, but the purpose is that some fetuses are less healthy. Realization of such criterion is the decision of the physician. 

Ease. This criterion is defined as "regarding the risk of reduction of the fetuses, when the above criteria are not met, the fetus that its reduction is easier will be chosen for the purpose of reduction". The assumption that the criterion of ease as an independent criterion comparing importance is not true. The reason is that the ease of reduction of one fetus over the other fetus in fact is the criterion of choosing the most important one. Therefore, we cannot think of the ease criterion as an independent criterion. This criterion depends on the specialist perspective. 

Random choice or parental rights. If the conditions are equal, choice of one fetus over the others is a preference without a logical reason. If the above conditions are not met, the physician or the parents can select the fetus for reduction just by random selection (lottery) or "absence of constraints rule". The meaning of "absence of constraints rule" is that in cases where no difference exists between two affairs and one is not preferred over the other and there is no possibility of collection of those two affairs, then the person who is obliged to choose is free to choose according to wisdom (16). 

The jurisprudence perspectives differ regarding random choice and picking up. According to recent perspective, the parents are free to choose the gender of the fetus, but It seems that random choice is more common among jurisprudences (34,36). Of note, the number of reductions is a specialized issue and the parents can only comment about the choice. Therefore, the parents’ demands for reduction of more fetuses to reach one fetus pregnancy is not acceptable. Of course, choosing according to gender can cause serious ethical and even legal challenges in the field of gender discrimination. In other countries, this issue is also considered and although the parents have the right to choose the gender of the fetus when they are deciding to reduce the number of fetuses, considering other discretions such as the necessity of gender balance in society is accounted as an obstruction for the right of choosing according to gender (37). 

Governmental criterion. Those previously mentioned criteria refer to the situations where the governor (religious ruling) did not order any writ. Protection of public rights makes governments ignore individual discretion. If the governor orders any writ, none of these criteria will be enforced. One example of such cases is that the number of genders is not equal. This issue causes many problems. Therefore, selecting gender to stabilize the society is chosen by the government (38). 

In cases that the doctor legally allowed elimination of one or more embryos in multiple pregnancies, deficiency in the fetuses (if one is defective) and importance (if there is any reason to prefer one fetus) can be criterions for selection of the goal of reduction. 

If the conditions of all fetuses are equal, the parents or the physician choose the fetuses by lottery or by picking them up (absence of constraints rule). Each perspective has its own devotees. Of note, if parents are permitted to choose, the recognition of the number of reductions is due to the physician. It seems that to prevent parents and physicians from misusing this situation in favor of single fetus pregnancy, passing an act by lawmakers is necessary. 

### Comparative study (Shiite perspective versus the UK law)

The first references to abortion in UK law appeared in the 13^th^Century. The law followed the Church’s teachings that abortion was acceptable until ‘quickening’ (which, it was believed was when the soul entered the fetus). In the Ellenborough Act (1803), the punishment of abortion after ‘quickening’ (i.e., when movement is felt at 16-20 weeks) was the death penalty although previously the punishment had been less severe. The legal situation remained the same for centuries. 

In 1861 (The Offences Against the Person Act), this penalty was reduced to life imprisonment. In 1929, Infant Life Preservation Act created a new crime of killing a viable fetus (at that time fixed at 28 weeks) in all cases except when the woman’s life was at risk. 

These laws caused thousands of women to resort to back-street abortions to prevent unwanted pregnancies or the need for abortions which led to permanent damage or death. For example, in 1923-33, 15% of maternal deaths were due to illegal abortions. In 1936, the Abortion Law Reform Association (ALRA) was established with the aim to campaign for the legalization of abortion. In 1967, the Abortion Act sponsored by David Steel, MP became a law and came into effect on April 27, 1968. This Act legalized abortion under certain conditions. In 1990, the 1967 Act was amended by the Human Fertilization and Embryology Act, which reduced the original time limit of 28 weeks to 24 weeks for most abortions (39). 

Currently, abortion is legal on a wide number of grounds in England, Wales, and Scotland since the Abortion Act of 1967, which is one of the most liberal abortion laws in Europe. Grounds for abortion under this Act include: i. Situations where the continuance of the pregnancy would involve risk to the life of the pregnant woman greater than if the pregnancy were terminated, ii. Termination is necessary to prevent grave permanent injury to the physical or mental health of the pregnant woman, iii. The pregnancy has not exceeded its 24^th^week and continuance of the pregnancy would involve risk, greater than if the pregnancy were terminated, of injury to the physical or mental health of the pregnant woman, iv. Pregnancy has not exceeded its 24^th^week and continuance of the pregnancy would involve risk, greater than if the pregnancy were terminated, of injury to the physical or mental health of any existing children of the family of the pregnant woman, v. There is a substantial risk that if the child were born it would suffer from physical or mental abnormalities as to be seriously handicapped, vi. To save the life of the pregnant woman, and vii. To prevent grave permanent injury to the physical or mental health of the pregnant woman (40). 

In 1999 approximately 8000 women who lived abroad travelled to England to have an abortion (41). The Law that regulates selective Reduction is contained in four separate statutes: Offences Against The Person Act 1861 (OAPA), Infant Life Preservation Act 1929 (ILPA), Abortion Act of 1967, and Human Fertilization and Embryology Act of 1990, section 37 (5) which amended the provisions of the Abortion Act sensibly to legalize the practice of selective reduction. With the exception of the 1990 Act, when these laws were framed, it was impossible to imagine that one or more fetuses might be destroyed, allowing others to survive to term. Section 58 of the OAPA prohibits the performance of an Act "with intent to procure the miscarriage of a woman with child" (42). 

In a comparison between the UK law and Shiite perspective, two points can be made. First, it is clear that in the UK the cases in which abortion is allowed are much more than allowed by Shia law. According to the Shia law, as previously mentioned, only the mother’s survival and fetus’s disability are the examples of harm that justify abortion. While in the first chapter of the Abortion Act of 1968 in the UK, mental or physical health of the mother or other children in the family are permissions for abortion. Generally it can be claimed that by the year 1929 the English parliament had a penal view toward abortion and the maximum protection was devoted to the fetus. However, the future laws, especially in 1990, legislations have preferred the mother’s life to the fetus. In Shia, the mother’s life preference over the fetus’s life is only legitimate when mother’s life would be at risk with continuation of pregnancy. Therefore, the Shia’s point of view is closer to the former English laws. The reason can be the effects of religion on these former regulations and the further amendments have followed the aim of decreasing illegal abortions and its fatal results. 

Second, despite the different perspective about abortion mentioned above, it is important to note that reduction of fetuses is specified in English law while Shia resources have not discussed it as a treatment. The authors of this article have attempted to determine the regulations of reduction in Shia books and their general principles and rules (such as abortion rules). Therefore, this issue should be studied by Shia in detail and the result should be passed as a law. However, no important contrast can be seen between Shia and English points of view about reduction. Two reasons prove this claim. First, treatment and saving other fetuses are the basic goals in reduction. Second, the main difference between Shia and English view in the field of abortion concerns the mother’s and other children’s mental and physical health. However mental and physical health has no place in reduction. 

## Discussion

Briefly, arbitrary choice is a strategy that the physicians suggest to eliminate the danger of multifetal pregnancy for mothers and the fetuses, that cause failure of the pregnancy. The aim of arbitrary choice is to reduce the number of fetuses to only one or two. Any order is not provided specifically about the criteria for embryo reduction in juridical sources. Therefore, it will inevitably be determined by the rules that govern abortion provisions or general juridical rules and principles which exist in Islamic sources (in primary and secondary ordinances). 

The primary ordinance of intentional death of a fetus in Shiite view is sanctity and its prohibition. However if there is an emergency, loss or hardship for the mother, the ordinance is secondary. As some jurists have stated, reduction of the fetuses before ensoulment of the fetus is permitted (10,14), but the authors believe that when the fetus endangers the mother’s life, there is no different between before and after ensoulment. 

The jurisprudence position in each example of fetus reduction defines reduction according to three premises: i. The conditions that multiple pregnancies will not entreat the mother or continuation of pregnancy and the fetuses are free of deficiency and there is no risk for them. In such cases reduction of embryos is forbidden, ii. Some of the fetuses are defective where, according to the secondary ordinance, reduction is permitted, and iii. Continuation of multiple pregnancies has risks for the fetuses. Such case raise the following assumptions: in a natural multiple pregnancy, if protection of the surplus fetuses endangers the life of all fetuses, we cannot act according to the lack of preference and cause the death of all fetuses. Therefore we should act according to an acceptable criterion. In artificial multiple pregnancies, the physicians are obliged to reduce the number of fetuses to avoid the situation of reduction, otherwise reduction of the fetuses is not permitted. If they do so, they will be prosecuted. 

A comparison between English and Shia rules about abortion and reduction indicates that in England abortion is more widely accepted and apart from mother survival or fetus disability, the mother’s or other children’s mental and physical health can justify abortion. However, the Shia consider abortion cases other than mother survival or fetus disability to be forbidden and taboo. Disregarding this difference, reduction by the aim of saving other fetuses is permitted in both systems and similarities can be seen in this field. 

## Conclusion

In an emergency situation, reduction of the fetuses is permitted. To choose the goal of reduction, the criterion is defection in fetuses or importance. The importance means if there is a reason to protect one fetus, (for example one of them is healthier); the other fetuses will be the goal of reduction. If the conditions are equal, the parents or the physicians can choose the fetus. It is noteworthy that if the choice of parents is permitted, choosing the number of them is due to the physician’s discretion. Finally the ordinance of the government and the existence of a rule are superior to other rules. 

Lack of any resource about various aspects of reduction (such as legal, moral and jurisprudence) is one of the most important limitations of this study. Therefore, an investigation into the relevant provisions of reduction in details such as civil liability of the physician and medical staff in embryo transfer or fetal reduction can be a suitable topic for future research. 
